# Volumetric thermometry in moving tissues using stack‐of‐radial MRI and an image‐navigated multi‐baseline proton resonance frequency shift method

**DOI:** 10.1002/mrm.70074

**Published:** 2025-09-10

**Authors:** Qing Dai, Shu‐Fu Shih, Omar Curiel, Tsu‐Chin Tsao, David S. Lu, Jason Chiang, Holden H. Wu

**Affiliations:** ^1^ Department of Radiological Sciences, David Geffen School of Medicine University of California Los Angeles Los Angeles California USA; ^2^ Department of Bioengineering University of California Los Angeles Los Angeles California USA; ^3^ Department of Mechanical and Aerospace Engineering University of California Los Angeles Los Angeles California USA

**Keywords:** liver ablation, motion compensation, MR thermometry, proton resonance frequency shift, radial MRI

## Abstract

**Purpose:**

To develop and evaluate a volumetric proton resonance frequency shift (PRF)–based thermometry method for monitoring thermal ablation in moving tissues.

**Methods:**

A golden‐angle‐ordered 3D stack‐of‐radial MRI sequence was combined with an image‐navigated multi‐baseline (iNAV‐MB) PRF method to reconstruct motion‐compensated 3D temperature maps with high spatiotemporal resolution and volumetric coverage. Two radial MRI reconstruction techniques, k‐space weighted image contrast filter (KWIC) and golden‐angle radial sparse parallel (GRASP) MRI, were implemented and compared within a sliding window reconstruction framework. Ex vivo motion phantom experiments were performed with high‐intensity focused ultrasound ablation to evaluate motion tracking and temperature accuracy using input motion waveforms and temperature probe readings as references. In vivo non‐heating swine experiments were conducted to assess temperature mapping stability in 3D liver regions of interest.

**Results:**

The proposed iNAV‐MB thermometry framework achieved volumetric coverage of 24 axial slices (3‐mm thickness), in‐plane resolution of 1.6 × 1.6 to 1.8 × 1.8 mm^2^, and effective temporal resolution of 0.98 s/volume. In ex vivo high‐intensity focused ultrasound experiments, motion tracking achieved correlation coefficients of 0.951 and 0.973, and temporal mean absolute errors were 1.80°C and 1.44°C using KWIC and GRASP, respectively. In vivo experiments demonstrated improvements in voxel‐wise temperature temporal SD from a median of 8.03 to 3.85°C (KWIC) and from a median of 7.23 to 2.37°C (GRASP), compared to single‐baseline PRF.

**Conclusion:**

The proposed stack‐of‐radial iNAV‐MB volumetric PRF thermometry framework can reliably track respiratory motion and map ablation‐associated temperature change. This framework has the potential to improve MRI‐guided thermal ablation in moving tissues.

## 1 INTRODUCTION

Thermal ablation is an increasingly utilized strategy for treating primary early‐stage liver tumors by raising the local tumor temperature to cytotoxic levels (above 50 to 60°C).^1^ Common energy sources for ablation include laser,^2^ high‐intensity focused ultrasound (HIFU),^3^ RF currents,^4^ and microwave fields.^5^ Thermal ablation is performed via minimally invasive approaches under image guidance and is considered the preferred approach for candidates not suitable for surgical resection.^6^ The technical success of thermal ablation procedures is highly dependent on reliable intraprocedural imaging for both guidance and monitoring to ensure treatment efficacy and safety.

Current standard imaging techniques for intraprocedural characterization of ablation include real‐time ultrasound^7^ and CT imaging,^8^ both of which track microbubble formation from water vapor during ablation. These microbubbles serve as a surrogate marker for the temperature distribution within the ablation zone. However, directly correlating microbubble formation to actual temperatures is challenging, which complicates the assessment of whether the ablation margin is sufficient to effectively treat the tumor.^6^, ^9^ Furthermore, it is unclear how close the ablation zone can approach critical structures, such as blood vessels or the liver capsule, before risking irreversible damage.^6^, ^9^

MR thermometry^10^, ^11^ is a powerful modality for monitoring thermal ablation. It provides noninvasive temperature mapping, enabling monitoring and prediction of heat‐induced tissue death and prevention of thermal damage to surrounding critical structures. Among various MR parameters sensitive to temperature change, the proton resonance frequency shift (PRF)–based thermometry method^12^, ^13^ is the most widely used. The resonance frequency of water protons decreases linearly with increasing temperature (approximately −0.01 ppm/°C),^12^, ^13^ a relationship that is largely tissue‐independent (except for adipose tissues, which lack hydrogen bonds). PRF thermometry has been successfully applied in monitoring liver tumor ablation using laser,^14^ RF currents,^15^ HIFU,^16^ and microwave therapies,^17^ and has been adopted clinically for MR‐guided minimally invasive transcranial procedures^18^ and for the treatment of localized breast^19^ and prostate cancers,^20^ bone metastases,^21^ and uterine fibroids.^22^

Despite the technical feasibility and promise, PRF thermometry faces significant challenges for monitoring liver tumor ablation because the presence of respiratory motion can cause artifacts that confound temperature measurements.^23^ Errors associated with intrascan motion can be partially mitigated using fast imaging sequences, such as single‐shot or segmented 3D EPI, or by implementing respiratory gating/triggering.^17^ Interscan motion‐induced PRF temperature errors can be addressed using a multi‐baseline approach^14^, ^24^ in which a library of baseline images at different motion positions is acquired prior to heating. During treatment, these baseline images can be compared with the incoming dynamic images based on the motion position derived from methods, such as navigator echoes^25^ or optical flow,^26^ to obtain a matched baseline image for calculating PRF thermometry. A recent review summarized common techniques for mitigating motion‐induced temperature artifacts.^27^

While the combination of fast imaging sequences and the multi‐baseline PRF approach has reduced motion‐induced temperature errors, most prior work has been limited to acquiring a single 2D slice or a few 2D slices.^24^, ^25^, ^26^, ^28^, ^29^ This limitation represents a tradeoff between spatiotemporal resolution, volumetric coverage, and SNR. In general, these constraints have restricted the ability of prior PRF methods to monitor a volume that encompasses the full tumor, 5 to 10 mm safety margins in all directions, and surrounding critical structures over the relevant range of respiratory motion. For effective monitoring of liver ablation procedures, achieving sufficient volumetric coverage is essential to ensure complete ablation to prevent local tumor recurrence and avoid thermal injury to critical structures.

Non‐Cartesian MR acquisition techniques, such as stack‐of‐radial MRI, offer a promising solution for monitoring ablation in moving tissues. Radial trajectories are inherently robust to motion due to their sampling geometry and repeated sampling of the k‐space center.^30^ Additionally, the golden‐angle‐ordered radial sampling strategy^31^ enables flexible reconstruction of dynamic images by re‐grouping of the data, allowing optimization of spatiotemporal resolution to improve volumetric coverage. Two radial reconstruction techniques, k‐space‐weighted image contrast (KWIC) filter^32^, ^33^ and golden‐angle radial sparse parallel (GRASP) MRI,^34^ are particularly suited for dynamic volumetric thermometry. KWIC reconstruction balances between effective temporal resolution and image fidelity by combining low‐frequency k‐space data from a narrow temporal window and high‐frequency data from a broader temporal window. KWIC reconstruction has been explored for volumetric PRF thermometry in static tissues and scenarios with limited motion,^35^, ^36^ but its performance in tissue undergoing continuous respiratory motion remains underexplored. GRASP reconstruction, on the other hand, exploits temporal sparsity and solves an optimization problem to reconstruct images from highly undersampled data. Whereas GRASP has been applied in various free‐breathing dynamic volumetric MRI applications,^34^, ^37^, ^38^ it has not yet been investigated in MR thermometry.

In this study, we propose a novel volumetric MR thermometry framework for monitoring thermal ablation in moving tissues, combining a golden‐angle‐ordered stack‐of‐radial MRI sequence with an image‐navigated multi‐baseline (iNAV‐MB) PRF method. The iNAV‐MB method enables direct motion estimation from the reconstructed dynamic magnitude images, facilitating motion‐compensated PRF temperature mapping. We implement and adapt two radial MRI reconstruction methods, KWIC^33^ and GRASP,^34^ with a sliding window‐based reconstruction approach for volumetric PRF thermometry. We compare KWIC + iNAV‐MB and GRASP + iNAV‐MB, as well as both KWIC and GRASP paired with conventional single‐baseline PRF thermometry, to evaluate their performance in measuring temperature changes during motion. The proposed framework is evaluated through an ex vivo tissue HIFU ablation study using a programmable motion phantom and in vivo non‐heating swine experiments.

## 2 METHODS

### 2.1 Overall framework for volumetric PRF thermometry

De Senneville et al. have previously discussed the general requirements for MR temperature mapping in monitoring thermal ablation.^11^ To obtain reliable volumetric thermometry in tissues experiencing respiratory motion (such as liver and kidney), several additional requirements should be met: (1) a FOV with sufficient volumetric coverage to capture most early‐stage tumors, target margin, and surrounding critical structures; (2) high effective temporal resolution to resolve intraframe motion caused by respiration, (3) robust motion compensation techniques to address interframe motion, and (4) minimal imaging artifacts arising from undersampling and motion effects within the tissue of interest. These considerations guided the development and evaluation of our proposed framework.

**FIGURE 1:**
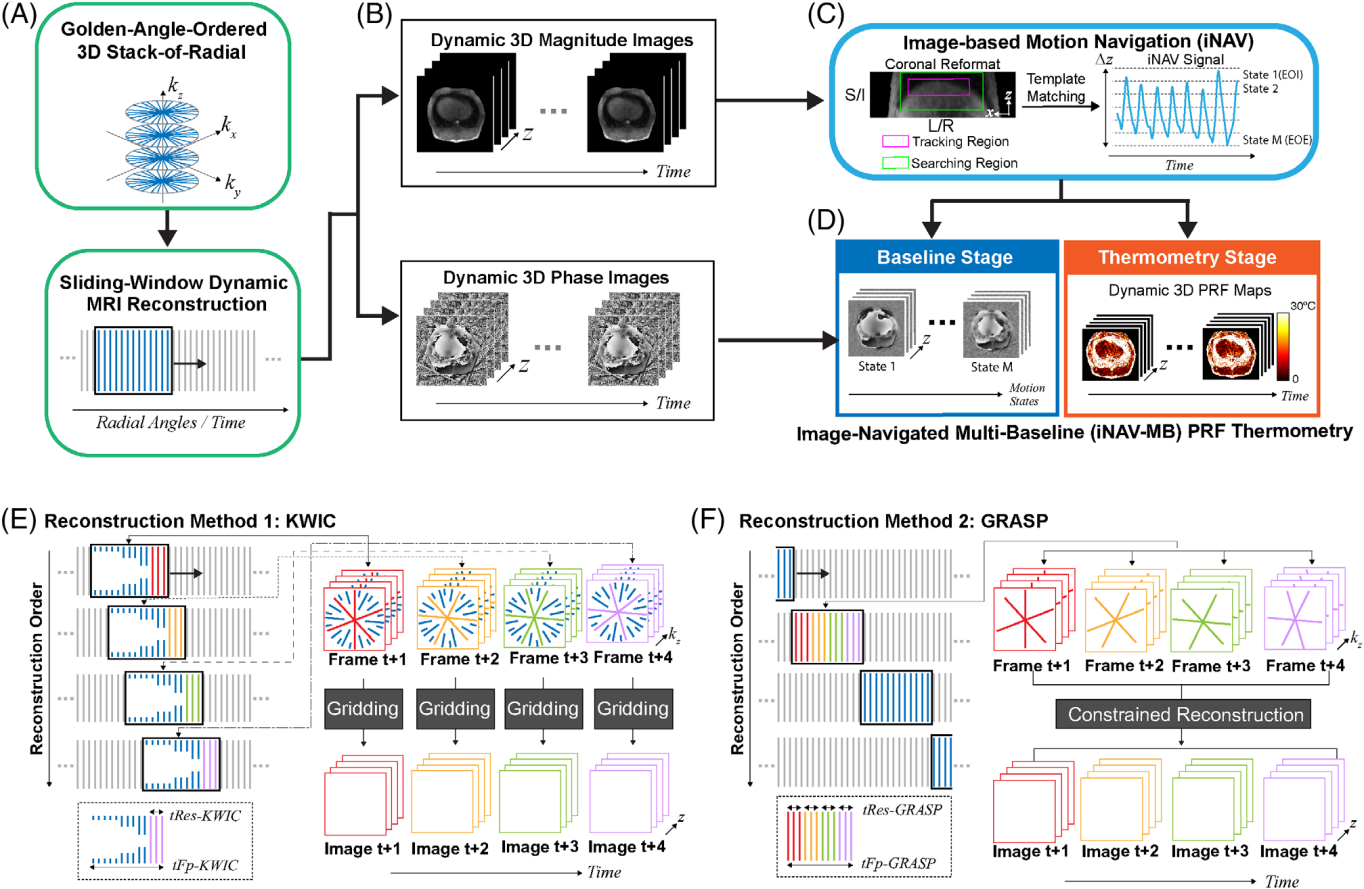
An overview of the proposed volumetric PRF thermometry framework in this paper. (A) The acquired dynamic multi‐echo golden‐angle 3D stack‐of‐radial data is reconstructed with a sliding‐window approach. (B–D) The reconstructed dynamic magnitude images are used to obtain the iNAV signal. A multi‐baseline library is formed using images from the baseline stage and the extracted iNAV signal. During the thermometry stage, the PRF temperature map at each time point is calculated with dynamic motion compensation (details are illustrated in Figure 2). (E, F) Diagrams of the two dynamic reconstruction methods used in this work. Their effective tRes and tFp are defined in the diagram and the text. EOE: end of expiration; EOI: end of inspiration; GRASP: golden‐angle radial sparse parallel MRI; iNAV, image‐based motion navigation; KWIC: k‐space weighted image contrast; PRF: proton resonance frequency shift; tFp, temporal footprint; tRes, temporal resolution.

The overall framework is presented in Figure 1. Data acquisition was performed using a golden‐angle‐ordered 3D stack‐of‐radial MRI sequence. This stream of radial k‐space data was reconstructed using a sliding window approach to form volumetric image frames with narrow temporal windows (i.e., high effective temporal resolution <1 s per volume). To achieve a narrow temporal window, each volumetric image frame required a high data undersampling factor compared to the Nyquist criteria (Figure 1A). Two dynamic radial MRI reconstruction techniques, KWIC and GRASP, were employed and compared. The reconstructed 3D magnitude images (axial volume) were reformatted into the coronal orientation, where image‐based motion navigation was used to extract a motion signal curve along the superoinferior (S/I) direction (Figure 1C). The reconstructed 3D phase images, combined with the extracted iNAV signals, were used to perform iNAV‐MB PRF thermometry, generating motion‐compensated 3D dynamic temperature maps (Figure 1D).

### 2.2 Golden‐angle‐ordered 3D stack‐of‐radial sequence

A 3D stack‐of‐radial multi‐echo spoiled gradient‐echo MRI sequence^36^, ^39^ was used. The sequence used radial sampling in the k_x_ and k_y_ directions and Cartesian sampling along the slice‐encoding (k_z_) direction. A bipolar multi‐echo readout gradient was used to acquire multi‐echo data. Data were first acquired along k_z_, using radial spokes with the same azimuthal angle, and subsequent radial data were acquired with an increment of a constant azimuthal angle of 111.25° derived from the golden ratio.^31^ This sampling scheme enables flexible adjustment of effective temporal resolution during the reconstruction of dynamic image frames. A multi‐echo acquisition was used to improve image SNR^35^ and enhance tissue boundary contrast^40^ for iNAV motion tracking (as described in section 2.4). Prior to image reconstruction, the multi‐channel radial k‐space data were prewhitened^41^ and corrected for gradient delays.^39^ An inverse Fourier transform was applied along the k_z_ direction to produce a set of 2D radial k‐space datasets corresponding to the multiple slices for subsequent reconstruction. Reconstructed dynamic 3D images were coil‐combined using adaptive coil combination.^42^ The multi‐echo magnitude images at each time point were echo‐combined using sum‐of‐squares for iNAV motion tracking, and the last echo phase image at each time point was used for PRF temperature mapping.

### 2.3 Dynamic radial MRI reconstruction

#### 2.3.1 KWIC reconstruction

KWIC uses radial k‐space data efficiently by including adjacent radial k‐space data from a larger temporal window for reconstruction, balancing between motion consistency and data undersampling. To capture the most recent information and minimize the temporal delay, a backward‐looking asymmetric KWIC filter^36^, ^43^ was implemented. For each KWIC reconstruction window, the innermost k‐space region contains only the most recently acquired radial data from a narrow temporal window, whereas the outer k‐space is filled with data from previously sampled radial k‐space data from a larger temporal window. Motion consistency is preserved because the central k‐space region contains data from a small acquisition window. The effective temporal resolution of the KWIC reconstruction window (*tRes‐KWIC*) is defined as the time required to acquire the innermost k‐space region from all k_z_ encodings,^33^, ^35^ whereas the temporal footprint (*tFp‐KWIC*) corresponds to the time required to acquire all radial data within a KWIC reconstruction window from all k_z_ encodings.^36^, ^43^, ^44^ The choice of effective temporal resolution (*tRes‐KWIC*) and temporal footprint (*tFp‐KWIC*) is optimized for motion tracking capability by lowering *tRes‐KWIC* and *tFp‐KWIC*, as recommended by Svedin et al.,^43^ while still maintaining a desired volumetric coverage and sufficient SNR. The effective temporal resolution of KWIC reconstruction (*tRes‐KWIC*) was chosen to be around 1 s per volume to resolve respiratory motion (around 3 to 5 s/breath), and the temporal footprint of KWIC reconstruction (*tFp‐KWIC*) was chosen to be around 10 s. 2D re‐gridding^45^ with density compensation^46^ was used for image reconstruction for each KWIC reconstruction window. The KWIC reconstruction window was advanced with a temporal step size that equals the number of radial angles filling the innermost k‐space region (Figure 1E).

#### 2.3.2 GRASP reconstruction

GRASP uses a compressed sensing framework with temporal sparsity constraints to handle highly undersampled radial data. Originally designed for dynamic contrast‐enhanced MRI, GRASP typically reconstructs data from the entire course of temporal contrast changes after the acquisition is completed. To adapt GRASP for dynamic temperature mapping, which requires continuously updated reconstruction results during a procedure for monitoring, we implemented a sliding‐window approach for GRASP to minimize the temporal delay, reconstructing only a few temporal frames at a time. For each GRASP reconstruction window, a total variation constraint was applied along the temporal dimension, and the following optimization problem was solved:

(1) $$\hat{m}=\underset{m}{\text{argmin}}{\left\|F\cdot S\cdot m-d\right\|}_{2}^{2}+\lambda {\left\|T\cdot m\right\|}_{1},$$

where F is the nonuniform fast Fourier transform operator, S is the coil‐sensitivity maps, m is the current set of dynamic 3D images to be reconstructed, d is the acquired multi‐echo multi‐channel 3D k‐space data, λ is the regularization parameter, and T is the temporal total variation operator. The effective temporal resolution of each frame in the GRASP reconstruction window (*tRes‐GRASP*) was defined as the time required to acquire radial angles from one temporal frame along all k_z_‐encoding steps, and the temporal footprint of each GRASP reconstruction window (*tFp‐GRASP*) was the time needed to acquire all radial angles within the reconstruction window. *tRes‐GRASP* and *tFp‐GRASP* were matched to those of KWIC reconstruction for comparison. The regularization parameter was determined using a grid search^34^ to achieve a balance between the motion averaging effects near tissue–air boundaries and radial undersampling artifacts. Coil sensitivity maps were estimated using radial data acquired during a non‐heating baseline stage. After each reconstruction step, the GRASP reconstruction window was advanced without overlapping with the previous one, using a temporal step size equal to the entire GRASP reconstruction window (Figure 1F). Both KWIC and GRASP reconstruction methods were performed offline using MATLAB R2021a (MathWorks, Natick, MA).

### 2.4 Image‐navigated multi‐baseline PRF thermometry

iNAV‐MB PRF thermometry combined image‐based motion tracking with multi‐baseline phase processing to minimize motion‐induced temperature errors. Magnitude images were used to estimate tissue displacement ∆z along the S/I direction at a time point t using a template‐matching algorithm.^47^ A rectangular region of interest (ROI) containing prominent tissue features (e.g., liver dome) was manually selected on a reference frame as the template for tracking, whereas a broader region that encompasses potential motion trajectories, as shown in Figure 1C, was automatically designated as the search region in subsequent image frames. The reference frame was specified to be a frame near the most stable motion position, for example, the end‐of‐expiration (EOE) position for the liver, during the non‐heating baseline stage. iNAV motion tracking was then performed automatically using least‐squares optimization to calculate the displacement of the template across time frames.

**FIGURE 2:**
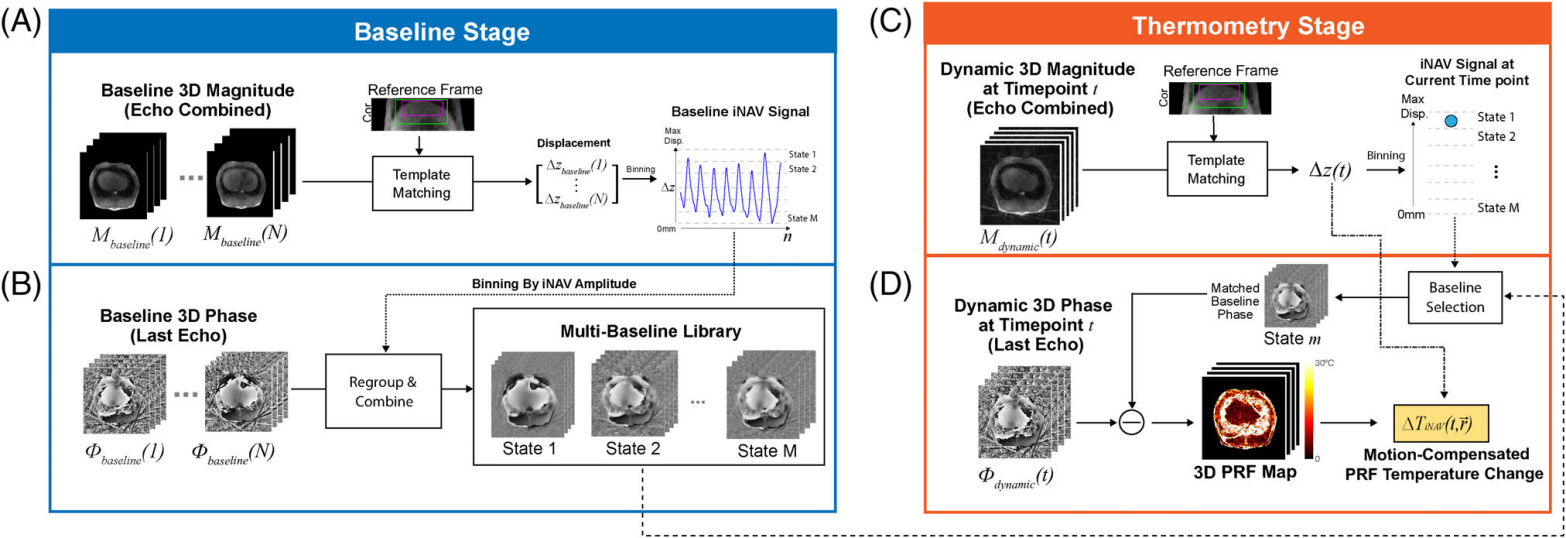
Detailed pipeline for the iNAV‐MB PRF thermometry technique. (A) During the baseline stage (prior to heating), the baseline magnitude images are echo‐combined, and the baseline iNAV signal is extracted using a template‐matching algorithm. A spatial region containing the liver dome from a baseline image at the EOE position is selected as the template. (B) Using the iNAV signal, the baseline phase images from the last TE are grouped and combined into different motion states and saved as a library. (C) During the thermometry stage, the liver displacement at each time point is calculated by comparing the incoming dynamic magnitude image to the template image. (D) Based on the liver displacement information, the corresponding baseline phase from the closest motion state is selected from the library and used to calculate PRF temperature maps.

After performing iNAV processing for all image frames in the baseline stage (Figure 2A), the phase images from the baseline stage were first sorted based on their iNAV signal and binned into M motion states, where M is determined as D/u, D represents the estimated S/I motion range in the subject (e.g., 15 to 20 mm), and u is the slice thickness (S/I resolution of the iNAV signal). Next, the baseline phase images with the same motion state were averaged, generating an iNAV multi‐baseline library containing high‐quality motion‐resolved baseline phase images representing each motion state (Figure 2B).

During the thermometry stage, similar iNAV processing was performed to estimate the iNAV signals ∆z(t) at each time point t (Figure 2C). Afterward, the optimal baseline image with a similar motion position was determined for calculating PRF temperature change maps (Figure 2D). The iNAV signal ∆z(t) was additionally used for computing motion‐compensated PRF temperature change ($∆T_{iNav}$) at each voxel $\vec{r}$ using the following equation:

(2) $$∆T_{\text{iNav}}\left(t,\vec{r}\right)=\frac{\phi_{d}\left(t,\vec{r}+∆z\left(t\right)\right)-{\bar{\phi }}_{m}\left(\vec{r}\right)}{\gamma \cdot \alpha \cdot B_{0}\cdot \mathrm{TE}},$$

where $\vec{r}$ is the voxel coordinate at (x,y,z), ${\bar{\phi }}_{m}$ is the 3D phase image from the multi‐baseline library (m=1,2,…M), $\phi_{d}$ is the dynamic 3D phase image at each time point, γ is the gyromagnetic ratio ≈42.58 MHz/T, α is the PRF temperature coefficient ≈−0.01 ppm/°C, $B_{0}$ is the main magnetic field strength, and TE is the echo time. In this way, the position of any tissue ROI within the imaging FOV can be continuously updated and compensated for each time point, ensuring accurate temperature monitoring in moving tissues. Field drift correction for the calculated temperature values was performed by using a first‐order polynomial fit to a non‐heated tissue region^48^ followed by temporal low‐pass filtering.^26^

### 2.5 Ex vivo motion phantom HIFU ablation experiment

**FIGURE 3:**
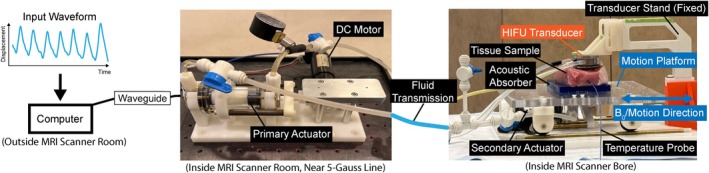
The setup of our ex vivo tissue motion phantom HIFU experiment. A custom MR‐compatible motion platform with a pair of hydrostatic actuators is used to emulate 1D respiratory motion along the $B_{0}$ direction. A motion waveform pre‐recorded from a healthy human subject with a peak‐to‐peak amplitude of 16 mm is used as the input. MR‐guided HIFU ablation is performed on a tissue specimen using an eight‐element transducer. The transducer position is fixed to the patient table while the tissue sample undergoes motion. Two fiber optic temperature probes are inserted into the tissue sample for temperature validation. The four‐channel flexible coil array (not shown) is placed on the HIFU transducer stand and does not move with the motion phantom. 1D, one‐dimensional; HIFU, high‐intensity focused ultrasound.

An ex vivo porcine tissue sample (pork chop) was allowed to reach room temperature before HIFU sonication. A programmable MR‐compatible motion phantom^49^ was used to evaluate the proposed framework, as shown in Figure 3. The phantom emulated one‐dimensional (1D) respiratory motion along the S/I direction using a waveform recorded from a previous human subject MRI experiment (peak‐to‐peak amplitude: 16 mm; respiratory period: 5.4 s/breath). The tissue sample was mounted on the motion platform. A research HIFU system (Image Guided Therapy, Bordeaux, France) was used. The HIFU transducer, which was fixed to the patient table, has an eight‐element annular array, an operating frequency of 1.5 MHz, maximum electrical power of 80 W, a diameter of 25 mm, a focal length of 20 mm, and a focal point size of 1 × 1 × 5 mm^3^. Under MR guidance, the HIFU focal point was positioned at the center of the tissue sample. Two 30 s HIFU sonication trials were performed prior to the main HIFU experiment to verify the position of the HIFU focal point. Two fiber‐optic probes (Micronor Sensors, Ventura, CA) were inserted into the tissue—one placed approximately 3 mm and the other 30 mm from the HIFU focal point—to monitor internal temperature and serve as reference temperature measurements. The positions of the temperature probe were verified using 3D Cartesian T_1_‐weighted gradient‐echo images (FOV = 278 × 191 × 66 mm^3^, resolution = 1.08 × 1.08 × 1.1 mm^3^, TE/TR = 2.58/4.19 ms). HIFU sonication was triggered at 30 s after the start of continuous MR acquisition and continued for 180 s at a constant electric power of 30 W.

Motion‐tracking accuracy was assessed by comparing the iNAV signals with input motion waveform using the Pearson correlation coefficient (CC). Temperature mapping accuracy was evaluated by calculating the temporal mean absolute error (tMAE) between the PRF‐based temperature measurements and the temperature probe readings. 3 × 3 ROIs surrounding the tips of the two temperature probes were selected and averaged for PRF calculation, and their S/I positions were dynamically updated using iNAV processing in Equation (2). Bland–Altman analysis and RMS error were used to assess the agreement between the PRF temperature measurements and probe readings during HIFU heating. Single baseline (SB) PRF thermometry was implemented using a single baseline phase image near the EOE position from the baseline library and compared with iNAV‐MB PRF.

### 2.6 In vivo non‐heating swine experiment

The animal study was approved by the University of California Los Angeles animal research committee (ARC 2022–074). Two Oncopig subjects with induced liver tumors^50^ (Duroc, male) and one healthy swine subject (Yorkshire, male), each weighing 30 to 40 kg, were used to evaluate the framework under non‐heating conditions and controlled respiratory cycles set at 5 s/breath. The swine subjects were sedated with intramuscular tiletamine hydrochloride–zolazepam hydrochloride (7 mg/kg, Telazol; Fort Dodge Laboratories, Fort Dodge, IA).

iNAV motion tracking was compared with the reference periodic signal pattern from mechanical ventilation. To evaluate temperature stability in a motion‐compensated, non‐heated region while avoiding potential motion artifacts from the tissue boundary, a 3D rectangular liver ROI with dimensions of 25 × 25 × 7 was manually selected within the homogenous region in the mid‐liver using the magnitude image at the EOE position and dynamically updated using iNAV processing in Equation (2). The tMAE and temporal SD (tSD) of the PRF‐based temperature were calculated on a voxel‐by‐voxel basis within the 3D ROI, relative to the expected temperature change of 0°C. The resulting voxel‐wise tMAE and tSD values were then averaged spatially across the ROI for each thermometry method. Both the ex vivo and in vivo experiments were performed on a 3 T MRI scanner (Magnetom Prisma, Siemens Healthineers, Erlangen, Germany) using a spine coil array and a four‐channel flexible coil array. The sequence and reconstruction parameters are listed in Table 1.

**TABLE 1:** Imaging parameters for the 3D stack‐of‐radial MRI thermometry technique used in ex vivo motion phantom HIFU ablation experiments and in non‐heating in vivo swine experiments. All experiments were performed at 3 T.

	Ex vivo motion phantom HIFU ablation	In vivo swine model
FOV (mm^3^)	230 × 230 × 72	300 × 300 × 72
Resolution (mm^3^)	1.79 × 1.79 × 3	1.56 × 1.56 × 3
Scan orientation	Axial	Axial
Flip angle (°)	9	9
TR (ms)	5.13	5.08
TE (ms)^a^	1.31/2.57/3.83	1.31/2.57/3.83
Bandwidth (Hz/px)	1130	1150
Total radial angles	3500	1500
Total scan time (min:s)	7:10	3:02
Reference respiration period (s/breath)	˜5.4	5
No. radial angles in center/ in total k‐space Region of KWIC filter	8/89	8/89
Effective temporal resolution/temporal footprint of dynamic PRF maps using KWIC reconstruction (s/volume)	0.98/10.96	0.98/10.85
No. radial angles in each/in total temporal frame(s) of GRASP reconstruction window	8/88	8/88
Effective temporal resolution/temporal footprint of dynamic PRF maps using GRASP reconstruction (s/volume)	0.98/10.83	0.98/10.73
Number of baseline images	20	30

^a^ The magnitude images from the three TEs were combined to improve SNR for image‐based motion navigation. The phase images from the last TE were used for temperature map calculation.

Abbreviations: GRASP, golden‐angle radial sparse parallel; HIFU, high‐intensity focused ultrasound; KWIC, k‐space weighted image contrast; PRF, proton resonance frequency shift; s, seconds.

## 3 RESULTS

### 3.1 Ex vivo motion phantom HIFU ablation experiment

**FIGURE 4:**
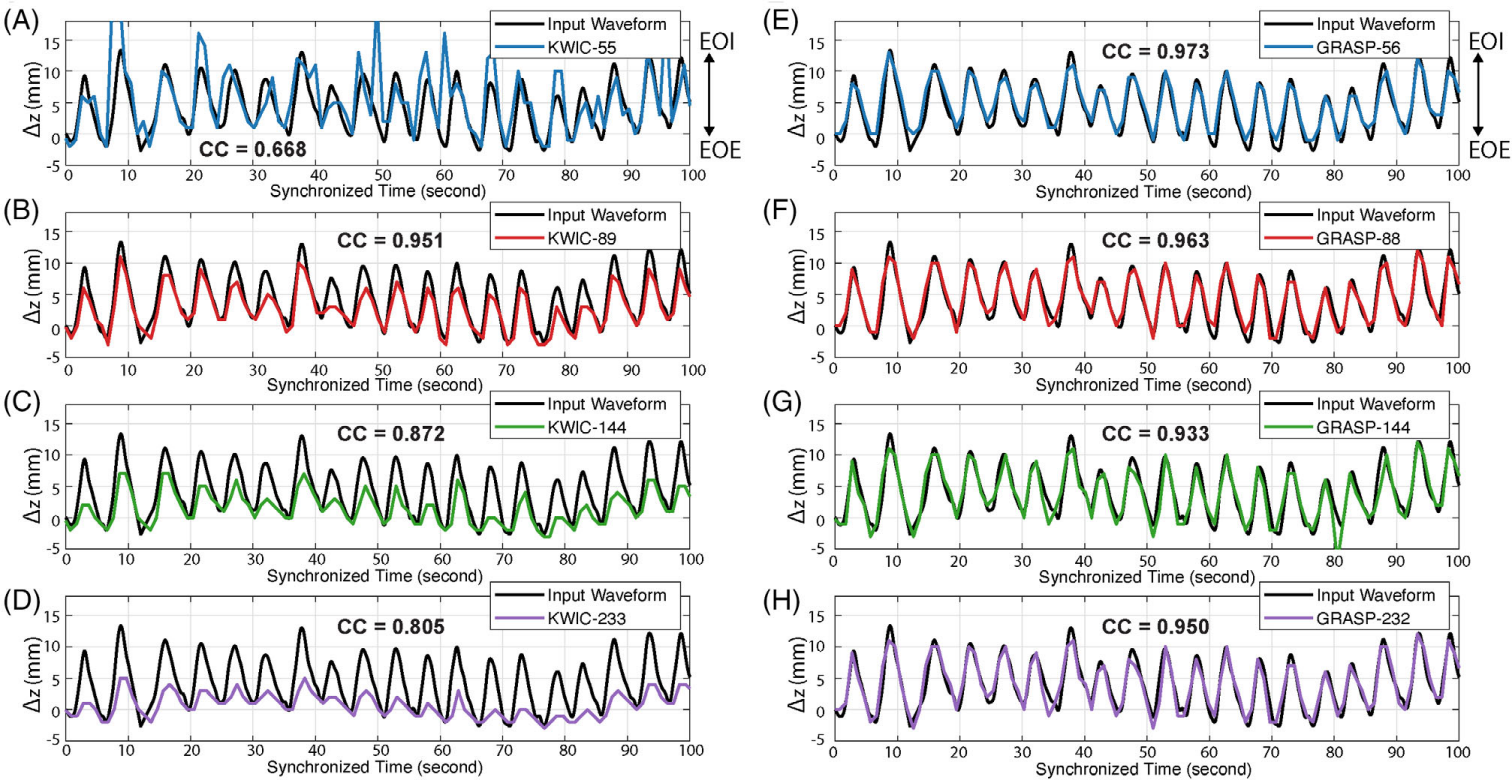
iNAV motion‐tracking results during the ex vivo motion phantom experiment compared to the reference input waveform. (A–D) iNAV motion‐tracking results using KWIC reconstruction with different number of radial angles that corresponds to KWIC temporal footprint (e.g., KWIC‐89), and (E–H) iNAV results using GRASP reconstruction with different number of radial angles that corresponds to GRASP temporal footprint (e.g., GRASP‐88). The number of radial angles that correspond to the effective temporal resolution was set to 8 for all cases. KWIC reconstruction needs to strike a balance between inaccurate motion tracking due to undersampling artifacts (i.e., in KWIC‐55) and a reduced range of motion (i.e., in KWIC‐233), likely due to temporal blurring associated with the use of a larger temporal footprint for the KWIC filter. On the other hand, GRASP reconstruction preserved temporal sharpness with different choices of temporal footprint. Pearson CC with respect to the input waveform is labeled in each of the plots. CC, correlation coefficient.

Figure 4 compares the extracted iNAV motion curves with the reference input waveform over 100 s of the ex vivo motion phantom HIFU ablation experiment. The same dataset was used to extract iNAV with two reconstruction methods, KWIC and GRASP, each with varying temporal footprints. Figure 4A–D shows the iNAV results using KWIC reconstruction by grouping 55 (KWIC‐55), 89 (KWIC‐89), 144 (KWIC‐144), and 233 (KWIC‐233) radial angles for each KWIC reconstruction window. Figure 4E–H shows the iNAV results using GRASP reconstruction by grouping 56 (GRASP‐56), 88 (GRASP‐88), 144 (GRASP‐144), and 232 (GRASP‐232) radial angles within each GRASP reconstruction window, selected to match the choice of KWIC temporal footprints for direct comparison. Both KWIC and GRASP reconstruction used the same effective temporal resolution of 0.98 s/volume (corresponds to eight radial angles to fill the central k‐space region). The iNAV curve calculates the relative motion displacement compared to a frame at the EOE position. The CC values between the iNAV motion curves and the input waveform are labeled in each subplot.

Among KWIC reconstruction results, KWIC‐89 provided the best tradeoff between motion fidelity and preserved motion range, achieving a CC = 0.951. In contrast, all GRASP reconstructions demonstrated consistently high motion‐tracking accuracy, with CC values exceeding 0.93. GRASP‐56 yielded the highest correlation (CC = 0.973), whereas GRASP‐88 achieved a comparable performance (CC = 0.963) and offered a temporal resolution most similar to KWIC‐89. Based on these findings, KWIC‐89 and GRASP‐88 were selected for temperature mapping and subsequent in vivo analysis.

**FIGURE 5:**
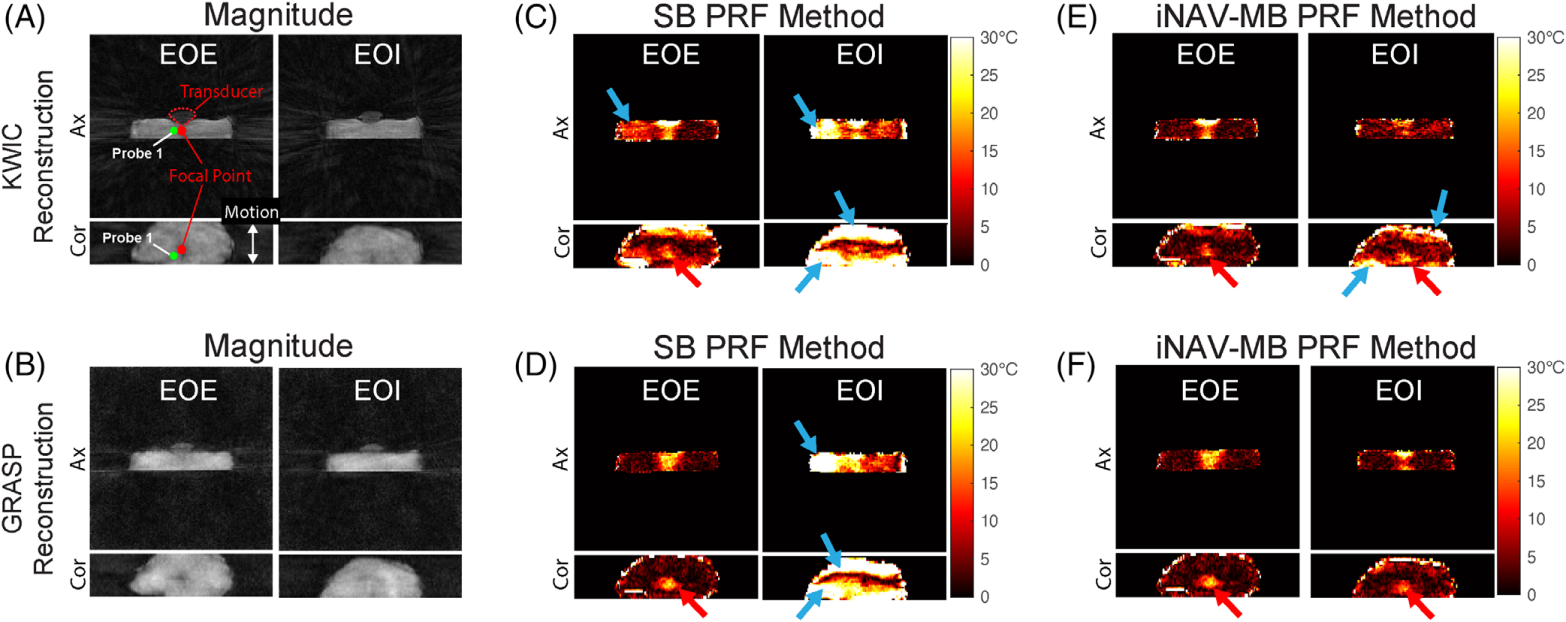
3D PRF absolute temperature change maps of the ex vivo motion phantom HIFU experiment using KWIC and GRASP reconstructions. (A, B) Reconstructed magnitude images from two frames corresponding to the EOE and EOI positions before the conclusion of HIFU ablation. (C, D) PRF temperature maps using a SB phase image. (E, F) PRF temperature maps using iNAV‐MB PRF thermometry consisting of six motion states. The white double‐arrows indicate the motion direction (along S/I direction). The green circle indicates the ROI placed at the approximate location of temperature probe 1 near the HIFU focal point. The proposed iNAV‐MB PRF method effectively suppressed motion‐induced temperature errors in SB PRF at EOI positions. Compared to KWIC reconstruction, the temperature maps from GRASP reconstruction yielded a sharper HIFU focal point at both EOE and EOI positions (red arrows) and less residual temperature errors in non‐heating regions (blue arrow). The backgrounds in PRF maps are masked out using their corresponding magnitude images. MB, multi‐baseline; SB, single baseline; S/I, superoinferior.

Figure 5 presents the PRF temperature mapping results during HIFU ablation. The multi‐baseline library consisting of *M* = 6 motion states was constructed from 20 dynamic images before HIFU heating. The proposed iNAV‐MB PRF method effectively reduced motion‐induced temperature errors compared to the SB PRF method at both the EOE and end‐of‐inspiration (EOI) positions. Compared to KWIC reconstruction, the temperature maps from GRASP reconstruction exhibited sharper definition of the HIFU focal point and had fewer residual errors in the non‐heating regions.

**FIGURE 6:**
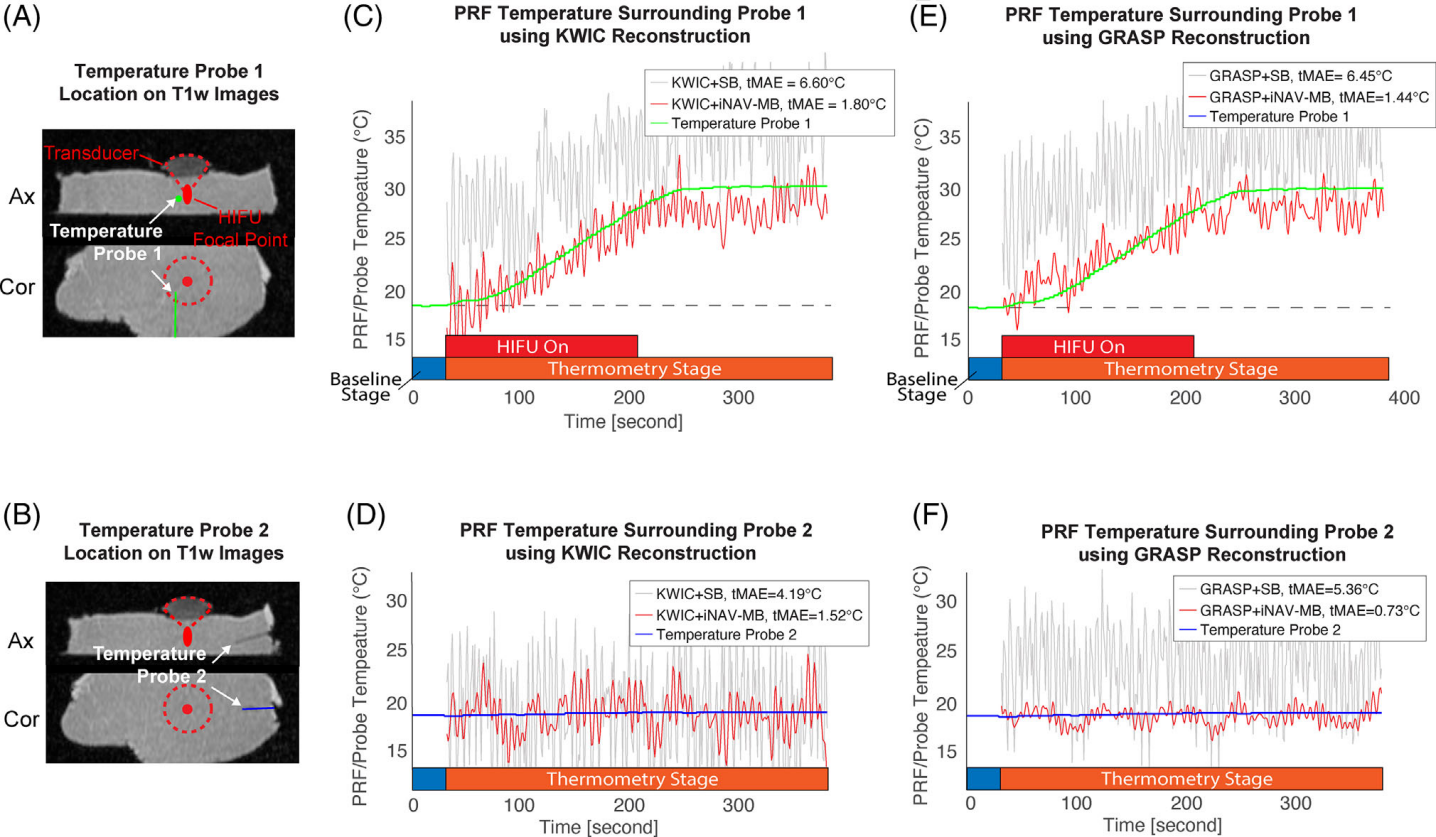
Results of the ex vivo motion phantom HIFU ablation experiment. (A–B) Locations of two temperature probes on T_1_w images. Probe 1 was placed approximately 3 mm away from the HIFU focal point, whereas probe 2 was placed in a non‐heating region approximately 30 mm away from the HIFU focal point. The HIFU focal point is labeled with red circles. (C–F) Temperature evolution from 3 × 3 ROIs extracted from the PRF temperature maps during and after the 180 s HIFU ablation. The ROI locations were dynamically updated using iNAV processing in Equation (2). The tMAE using the iNAV‐MB PRF method relative to the temperature probe readings demonstrated a marked improvement in temperature accuracy compared to the SB PRF method. T_1_w, T_1_‐weighted; tMAE, temporal mean absolute errors.

The current version of our 3D stack‐of‐radial thermometry research sequence supports a maximum of 3500 acquired radial angles and therefore we focused on the HIFU heating and the initial cooldown period for PRF thermometry evaluation. Figure 6 shows the PRF temperature change evolution and temperature probe readings over a 350 s time span. Compared to the temperature probe readings, KWIC reconstruction achieved a tMAE of 1.80°C in the heating region, whereas GRASP reconstruction achieved a tMAE of 1.44°C. In non‐heating regions, the tMAE was 1.52°C and 0.73°C for KWIC and GRASP reconstructions, respectively. Note that the brief temperature “plateau” observed immediately after HIFU sonication in Figure 6C,E can be attributed to the heat diffusion from the HIFU focal point because both the PRF ROI and temperature probe were positioned adjacent to (but not directly at) the HIFU focus. For reference, the extended temperature evolution recorded by temperature probes is shown in Figure S1. Figure S2 presents the Bland–Altman plots, where the mean differences between PRF thermometry and temperature probe readings during HIFU heating were −0.47°C and 0.39°C for the KWIC + iNAV‐MB and GRASP + iNAV‐MB methods, with corresponding RMS error values of 1.67°C and 1.70°C, respectively.

### 3.2 In vivo non‐heating swine experiments

**FIGURE 7:**
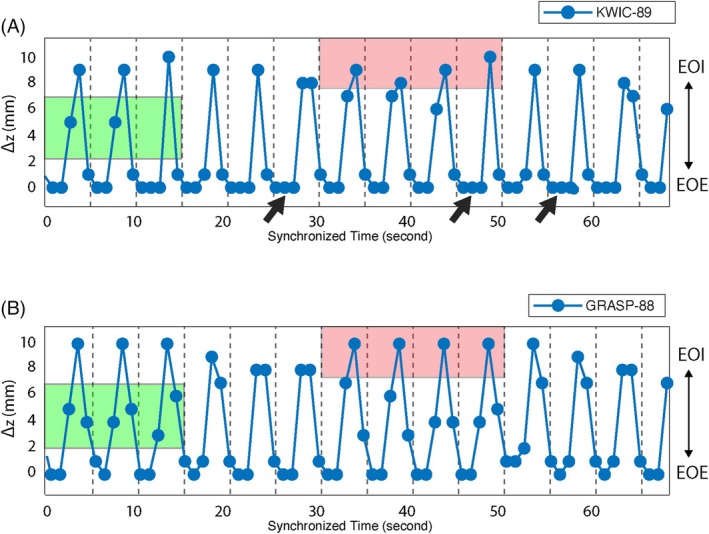
iNAV motion tracking results from an in vivo swine subject under controlled ventilation with (A) KWIC reconstruction with 89 radial angles within each reconstruction window and (B) GRASP reconstruction with 88 radial angles within each reconstruction window. Gray vertical lines indicate the reference respiratory period set at 5 s/breath. GRASP reconstruction resolved more distinct motion positions (green shaded region) and a larger range of motion (red shaded region) compared to KWIC reconstruction. The iNAV results from KWIC exhibited “plateaus” at EOE (black arrows), likely due to the motion averaging effects.

Figure 7 shows the extracted iNAV motion curves from a swine subject under controlled ventilation. The same dataset was reconstructed using KWIC‐89 and GRASP‐88, respectively, to generate the iNAV signals. Both methods captured the periodic breathing pattern, consistent with the expected 5 s respiratory cycle. GRASP reconstruction resolved more distinct motion positions, with clearer transitions between the end‐of‐inspiration and EOE positions, whereas KWIC tended to average between these positions, likely due to temporal smoothing. GRASP also exhibited a slightly larger motion range in the S/I direction, although this difference with KWIC was less pronounced than in the ex vivo experiment.

**FIGURE 8:**
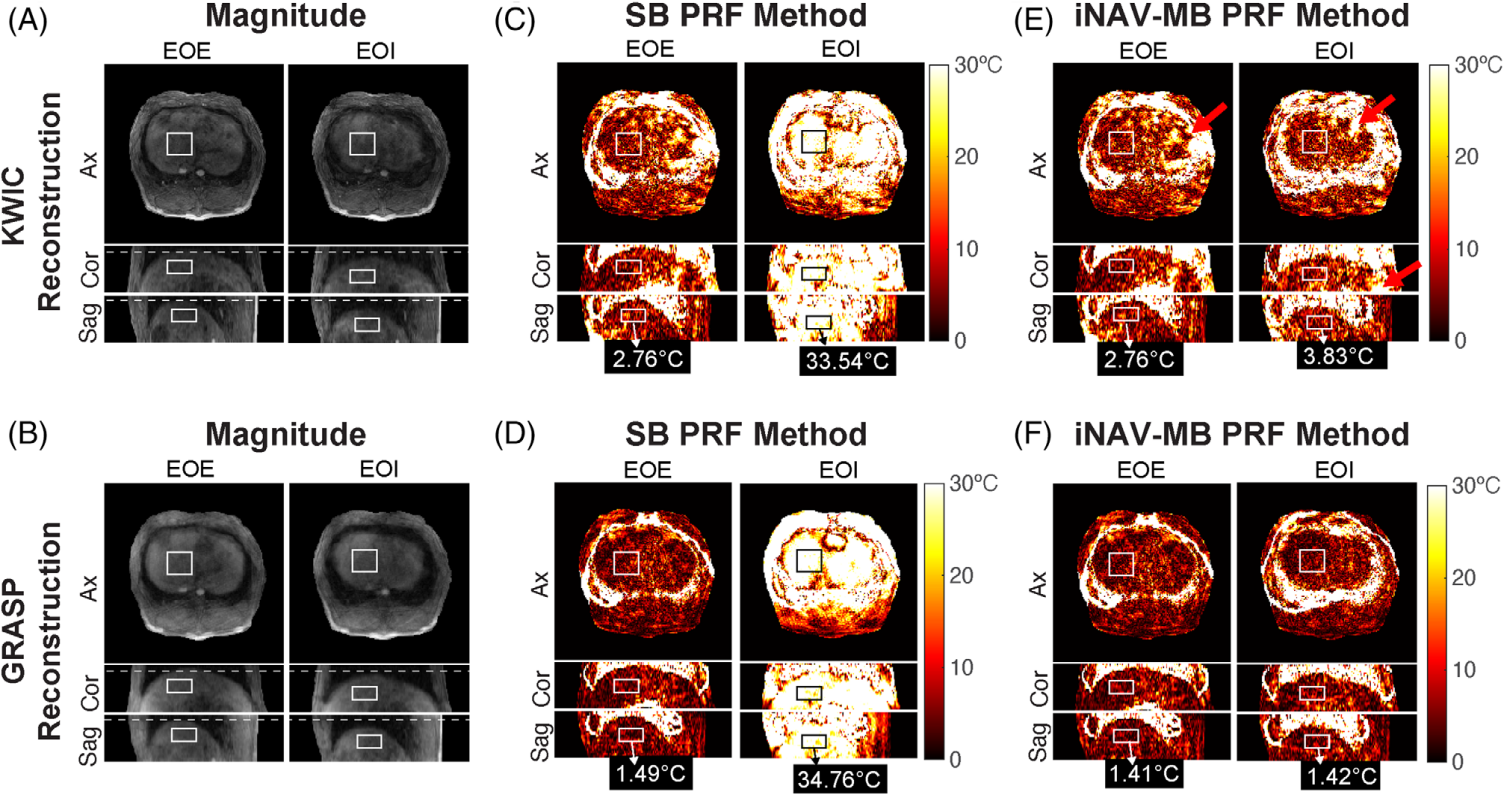
3D PRF absolute temperature change maps from a representative swine subject (same as in Figure 7) during the in vivo non‐heating experiment. (A, B) Reconstructed magnitude images from two selected frames corresponding to the EOE and EOI positions using KWIC and GRASP reconstruction. (C, D) PRF temperature maps using a SB phase image from EOE position and (E, F) using iNAV‐MB PRF methods with six motion states. Residual temperature errors are present in KWIC reconstruction results (red arrows). The white‐and‐black rectangular ROIs indicate a 3D liver ROI with size 25 × 25 × 7 pixels. The spatially averaged mean absolute temperature errors in this 3D volume at each frame are reported at the bottom of the temperature maps, relative to the expected temperature change of 0°C (no heating). The positions of the 3D ROIs were dynamically updated continuously using iNAV processing, and their locations were dynamically updated using Equation (2) (see Video S1). White dashed lines indicate reference liver positions.

Figure 8 shows the temperature mapping results using a baseline library consisting of 30 dynamic images under non‐heating conditions from the same swine subject in Figure 7. The proposed iNAV‐MB PRF method effectively suppressed motion‐induced temperature errors within the liver volume compared to the SB PRF method. Compared to the PRF maps from KWIC reconstruction, those from GRASP reconstruction exhibited less temperature variation in the liver, where no heating was expected. Video S1 demonstrates the capability of dynamically updating the position of a 3D liver ROI during motion by using the iNAV motion signals.

Across all three swine subjects, the tSD for KWIC reconstruction decreased markedly from a range of 7.13 to 8.19°C (median of 8.03°C) with the SB PRF method to 3.74 to 3.86°C (median of 3.85°C) with the iNAV‐MB PRF method. Similarly, the tSD for GRASP reconstruction was substantially reduced from 4.77 to 7.82°C (median of 7.23°C) with the SB PRF method to 1.76 to 2.43°C (median of 2.37°C) with the iNAV‐MB PRF method. Key results from the in vivo non‐heating swine experiments are summarized in Table 2.

**TABLE 2:** Summary of the temperature mapping results from the in vivo non‐heating swine experiments.

	Swine subject 1		Swine subject 2		Swine subject 3	
	tMAE(°C)	tSD(°C)	tMAE(°C)	tSD(°C)	tMAE(°C)	tSD(°C)
KWIC + SB	28.99	8.03	9.72	8.19	13.95	7.13
GRASP + SB	28.31	7.82	9.33	7.23	13.05	4.77
KWIC + iNAV‐MB	3.33	3.74	3.45	3.86	3.25	3.85
GRASP + iNAV‐MB	**2.50**	**2.37**	**2.09**	**2.43**	**1.48**	**1.76**

*Note*: The tMAE and tSD from the dynamically updated 3D ROIs (as shown in Figure 8) were computed on a voxel‐by‐voxel basis, and the spatial averages across the ROI were reported for each thermometry method. Bold font indicates the temperature mapping method with the best performance.

Abbreviations: iNAV‐MB, image‐navigated multi‐baseline PRF method; SB, single baseline PRF method; tMAE, temporal mean absolute error; tSD, temporal SD.

## 4 DISCUSSION

This work developed a stack‐of‐radial MRI‐based approach for rapid volumetric thermometry in moving tissues, such as liver, achieving continuous 3D temperature mapping with 24 axial slices (3‐mm thickness), in‐plane resolution of 1.6 × 1.6 to 1.8 × 1.8 mm^2^, and an effective temporal resolution of 0.98 s/volume. To address the motion‐induced baseline mismatch, we proposed an image‐navigated multi‐baseline technique, or iNAV‐MB, to directly estimate and correct motion during multi‐baseline PRF thermometry. Two radial MRI reconstruction methods, KWIC and GRASP, were implemented and adapted for dynamic PRF thermometry using a sliding window approach. We compared the combination of KWIC and iNAV‐MB to the combination of GRASP and iNAV‐NB and evaluated their performance in measuring temperature changes during respiratory motion. The performance of these two approaches was validated through an ex vivo tissue motion phantom HIFU ablation experiment and achieved a temperature tMAE of <1.5°C during HIFU ablation compared to the temperature probe readings. For in vivo non‐heating swine experiments, we achieved temperature mapping precision in terms of tSD of 3.74 to 3.86°C and 1.76 to 2.43°C for the combination of KWIC + iNAV‐MB and GRASP + iNAV‐MB, respectively, within motion‐compensated 3D liver ROIs across three swine subjects.

### 4.1 Stack‐of‐radial MRI‐based volumetric thermometry

Stack‐of‐radial MRI offers an advantageous solution for volumetric PRF thermometry in moving tissues due to its inherent robustness to motion and flexibility in balancing spatiotemporal resolution. Whereas prior studies have demonstrated the feasibility of stack‐of‐radial–based PRF thermometry in largely static organs^35^, ^36^ (i.e., brain, breast, prostate), our work successfully extended this technique to abdominal organs, such as the liver, in the presence of continuous respiratory motion. GRASP, on the other hand, had not been previously investigated for PRF temperature mapping.

In contrast to EPI‐based thermometry methods,^24^, ^25^, ^26^, ^28^, ^29^, ^51^, ^52^ which are typically constrained to 2D acquisitions or limited slice coverage, stack‐of‐radial acquisitions enable true 3D temperature monitoring. A recent EPI‐based study extended slice coverage using simultaneous multislice acquisition with multiband acceleration, achieving 12‐slice coverage at 700 ms per volume in a moving phantom.^53^ However, higher multiband factors degraded imaging quality due to aliasing. Our stack‐of‐radial approach overcame this limitation by supporting higher undersampling factors—up to 25‐fold ex vivo and 38‐fold in vivo based on Nyquist criteria—while maintaining image fidelity, as demonstrated by clear delineation of tissue boundaries and ablation zones (Figures 5 and 8). This framework enabled dynamic 3D temperature mapping across 24 axial slices and around 1 s effective temporal resolution per volume, a substantial advance in volumetric coverage for dynamic PRF thermometry in moving tissues. While the TE (TE = 3.83 ms) that we used for thermometry was shorter than those typically used for maximizing temperature‐to‐noise ratio, it was chosen to prioritize temporal resolution and volumetric coverage, both critical for monitoring thermal therapies in moving abdominal organs. Further improvements in volumetric coverage or temporal resolution may be possible by incorporating more efficient sampling strategies such as stack‐of‐radial with k_z_ undersampling^54^ or stack‐of‐radial EPI hybrid techniques.^55^

Our swine study was designed to closely mimic the clinical setup, including coil placement, use of general anesthesia, and respiratory cycle duration.^56^ Therefore, a similar level of temperature mapping performance is expected when translating to human subjects. However, recalibration of KWIC and GRASP reconstruction parameters will be necessary to accommodate differences in respiratory patterns, body size, and tissue composition.

### 4.2 Advantages of iNAV‐MB

We developed an image‐navigated multi‐baseline PRF thermometry method that leveraged the reconstructed magnitude images to guide multi‐baseline temperature mapping. Whereas previous motion navigation methods for PRF thermometry often relied on separate 1D or 2D navigator echo acquisitions,^47^, ^57^ our iNAV approach extracted motion directly from the magnitude image data itself, enabling motion self‐navigation without the need for additional acquisition modules. In our iNAV approach, motion was estimated by comparing dynamic magnitude images to a baseline frame using a region containing distinct features to achieve frame‐to‐frame tissue displacement tracking. A multi‐echo acquisition was used to improve magnitude image SNR and enhance tissue boundary contrast for robust navigation. The extracted iNAV motion information was used throughout the multi‐baseline PRF thermometry pipeline for baseline image sorting, dynamic image matching, and target ROI tracking. Compared to a previous trajectory‐based navigator‐free method proposed by Svedin et al.,^35^ which relied on pseudo‐golden‐angle matching of k‐space data for implicit motion correction, our approach directly estimated motion displacement to enable explicit, active motion tracking, which is a requirement for applications such as HIFU beam steering^58^ or robotic‐assisted interventions.^59^

Another major advantage of our iNAV‐MB method is that it enables respiratory motion tracking in the dominant S/I direction without requiring acquisition in specific planes, whereas previous multi‐baseline techniques were limited to sagittal or coronal acquisitions.^24^, ^51^, ^52^ This flexibility is enabled by the high effective temporal resolution and volumetric coverage of the stack‐of‐radial sequence. As validated in the motion phantom experiment (Figure 4), iNAV‐MB achieved high motion‐tracking accuracy under a variable breathing pattern. This enabled continuous temperature mapping across all motion states, with markedly improved temperature stability and reduced errors compared to single‐baseline PRF. Nevertheless, the single‐baseline approach using GRASP demonstrated good temperature stability when the motion position matched the baseline phase image (Figures 5D and 8D). Therefore, a motion‐gated approach, such as using a data acceptance window at EOE, could be a practical alternative when monitoring a specific motion state is preferred, albeit at the cost of skipping time frames at other motion states. Although this study focused on tracking motion in the S/I direction, the proposed framework can be extended to incorporate anteroposterior and left–right motion components in future implementations to enable comprehensive 3D motion tracking.

### 4.3 KWIC versus GRASP reconstruction

KWIC and GRASP reconstruction techniques employ different strategies in how they fill in k‐space and handle temporal information, leading to differences in motion tracking fidelity and PRF temperature mapping accuracy. KWIC reconstruction uses a k‐space filtering strategy for which the low‐frequency central k‐space region, which is critical for overall image contrast and contains slow‐changing spatial temperature information, is updated frequently using data from a narrow temporal window. Meanwhile, the high‐frequency peripheral k‐space regions, which capture finer structural details and rapidly changing spatial features, are filled with a broader set of spokes collected over a longer period. This filtering strategy helps suppress undersampling artifacts (Figures 5A and 8A), capture overall motion periodicity (Figure 4A–D), and measure the temperature changes (Figure 6C). We implemented a backward‐looking KWIC filter configuration to ensure a timely update of the central k‐space region. However, KWIC filtering will introduce averaging effects due to a mixing of motion and temperature information in the peripheral k‐space regions, especially if the temporal footprint is not carefully optimized. As shown in Figure 4A–D, broader temporal footprints led to more severe averaging effects with reduced apparent tissue displacement, whereas a shorter temporal footprint improved motion fidelity but increased the risk of errors resulting from undersampling artifacts. These effects can also degrade temperature accuracy, especially when thermal gradients evolve quickly in the tissues. To balance these effects, we identified an optimal configuration for our experimental data using KWIC with eight radial angles in the center of k‐space and 89 radial angles in the full reconstruction window and achieved a motion‐tracking CC of 0.951 relative to the input waveform and a temperature accuracy of 1.80°C compared to reference probe data. Such motion averaging effects were less pronounced during in vivo experiments with mechanical ventilation, likely due to higher SNR and more regular breathing patterns compared to the phantom study. These results suggest that, although KWIC offers fast, real‐time compatible reconstructions, its use should be cautioned in scenarios requiring more precise motion and temperature monitoring in which averaging effects may lead to underestimation of temperature rise or tissue displacement amplitude.

In contrast, GRASP uses a compressed sensing reconstruction framework that balances data consistency and temporal sparsity along the temporal direction. The golden‐angle‐ordered radial sampling trajectory provides flexible k‐t sampling to support arbitrary temporal grouping as well as incoherent undersampling artifacts. GRASP jointly reconstructs several temporal image frames within a single reconstruction window without explicit data sharing between the frames, maintaining data fidelity and temporal stability in low‐ and high‐frequency k‐space regions. This approach avoids the data mixing effects inherent to KWIC and enables reconstruction of the full‐resolution k‐space data, making it suitable for both motion tracking and dynamic temperature mapping, even at high undersampling rates. In this work, GRASP reconstruction demonstrated excellent motion tracking accuracy across a range of temporal footprints (Figure 4E–H), highlighting its robustness to averaging effects that were associated with KWIC. In the in vivo experiments, GRASP also resolved more distinct motion positions and preserved a wider motion range compared to KWIC, as shown in Figure 7, consistent with the trends observed in the ex vivo experiments. Moreover, GRASP achieved higher temperature accuracy during the ex vivo experiment in both heating and non‐heating regions, with a tMAE of 1.44°C and 0.73°C, respectively. In contrast, KWIC yielded higher tMAE values of 1.80°C and 1.52°C in the same regions. In the in vivo experiments, GRASP demonstrated improved temperature mapping performance in both tMAE and tSD compared to KWIC. This higher precision in temperature mapping could enable more accurate cumulative thermal dose estimation, which is critical for assessing treatment outcomes in thermal ablation. However, GRASP's main drawback remains the intensive computational cost. Our implementation required approximately 62.5 s to reconstruct a single 2D slice from one reconstruction window, limiting its use for real‐time applications. In addition, GRASP's temporal update step size equals the full span of the GRASP reconstruction window (as shown in Figure 1F), resulting in the generation of several time frames at once but at a slower update step size of 10 s using the parameters listed in Table 2. In contrast, KWIC reconstruction does not require an iterative approach and enables more frequent updates because its temporal update step size depends only on the radial spokes in the central k‐space region (as shown in Figure 1E). The ability to update more frequently makes KWIC more suitable for scenarios in which immediate temperature feedback is required. Reducing the GRASP temporal footprint (e.g., using GRASP‐56 instead of GRASP‐88) may slightly shorten reconstruction time by lowering the number of k‐space samples; however, the overall latency remains primarily dominated by the iterative optimization process. Future studies should investigate optimizing GRASP parameters, such as using shorter temporal footprints and fewer spokes to enable more frequent updates without compromising temperature mapping performance. Accelerated reconstruction techniques, including GPU‐based implementations^60^ or deep learning approaches,^61^, ^62^ may enhance the feasibility of 3D non‐Cartesian stack‐of‐radial MRI for real‐time volumetric temperature monitoring.

### 4.4 Limitations and Future Directions

There are limitations to this study. First, although the current implementation of KWIC and GRASP reconstruction utilized GPU‐based nonuniform fast Fourier transform^63^ to accelerate radial MRI reconstruction, the processing was performed offline in a retrospective manner and is not yet ready for real‐time temperature monitoring. Further improvements are needed in optimized software implementation^64^ and integration with online reconstruction frameworks such as Gadgetron.^65^, ^66^ Second, the current iNAV‐MB method measures rigid body motion along the S/I direction. While this strategy was effective for prerecorded breathing patterns from healthy human subjects and controlled ventilation in swine, more complex motion patterns, such as nonrigid deformation or irregular breathing, may require more comprehensive motion‐compensation techniques, such as nonrigid motion estimation^67^, ^68^ or incorporation of anteroposterior and left–right motion components.^69^ Third, this study validated the proposed thermometry framework through a single ex vivo HIFU ablation experiment with a single respiratory waveform. Future studies should evaluate performance under a wider range‐of‐motion patterns and ablation protocols, as well as in vivo ablation to better establish generalizability for clinical translation.

## 5 CONCLUSION

We developed and validated a volumetric MR thermometry framework for moving tissues based on a golden‐angle‐ordered stack‐of‐radial 3D MRI acquisition combined with dynamic radial reconstruction using KWIC and GRASP, image‐based motion tracking, and multi‐baseline PRF thermometry. This approach enables high spatiotemporal resolution and motion‐compensated 3D temperature monitoring in moving tissues, offering marked improvement in volumetric coverage over conventional methods. The proposed volumetric thermometry framework can be a potential solution for improving the accuracy and robustness of MR‐guided thermal ablations in moving organs.

## Supporting information


**Figure S1.** Temperature probe readings during the full ex vivo motion phantom experiment, including an extended cooldown period. The probe locations are shown in Figure 6A,B. PRF temperature measurements during the thermometry stage are plotted for reference.
**Figure S2.** Bland–Altman plots comparing PRF thermometry measurements using (A) KWIC+ iNAV‐MB and (B) GRASP + iNAV‐MB at ROI 1 near the HIFU focal point (see Figure 6A) with respect to reference temperature probe readings. The mean difference (MD) and 95% limits of agreement (LoA) are labeled in the plots. In addition, RMS error values are reported.

**Video S1.** Video display of the reconstructed 3D dynamic PRF temperature change maps during in vivo non‐heating swine experiments. The results from KWIC (top row) and GRASP reconstruction (bottom row) are compared. The position of a rectangular ROI (white box) was continuously updated using the iNAV signals. White dashed lines indicate the reference liver position. Green dashed lines indicate a dynamically updated axial plane based on the iNAV processing along the S/I direction.
