# Supplementary material for: Volumetric thermometry in moving tissues using stack‐of‐radial MRI and an image‐navigated multi‐baseline proton resonance frequency shift method
Source: Magn Reson Med. 2025 Sep 10;95(2):803–19. doi: 10.1002/mrm.70074 (PMC12681303; doi:10.1002/mrm.70074)
Supplement: Supplementary file 1 — Figure S1. Temperature probe readings during the full ex vivo motion phantom experiment, including an extended cooldown period. The probe locations are shown in Figure 6A,B. PRF temperature measurements during the thermometry stage are plotted for reference. Figure S2. Bland–Altman plots comparing PRF thermometry measurements using (A) KWIC+ iNAV‐MB and (B) GRASP + iNAV‐MB at ROI 1 near the HIFU focal point (see Figure 6A) with respect to reference temperature probe readings. The mean difference (MD) and 95% limits of agreement (LoA) are labeled in the plots. In addition, RMS error values are reported. [file MRM-95-803-s001.docx]

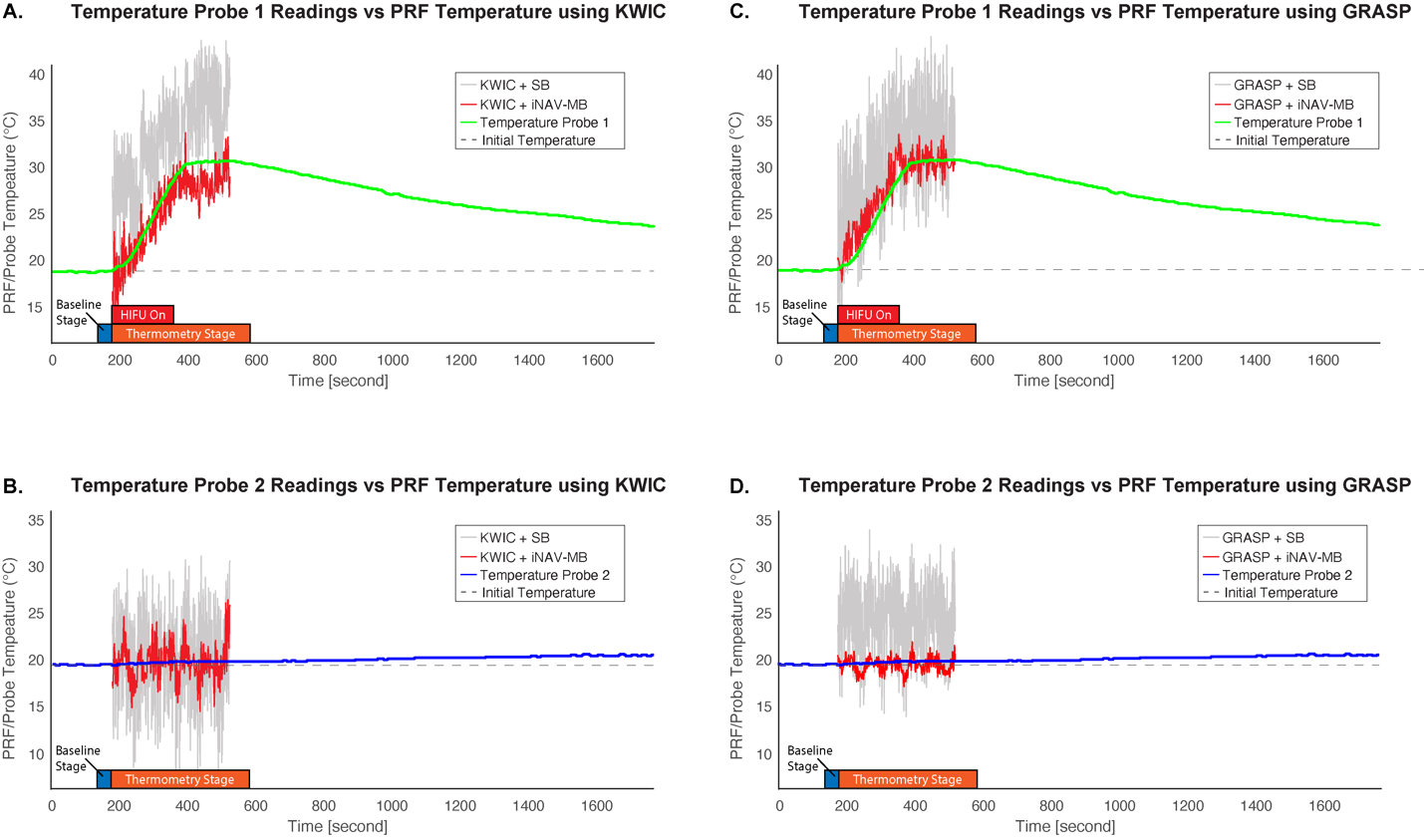


**Supporting Information Figure S1.** Temperature probe readings during the full ex vivo motion phantom experiment, including an extended cooldown period. The probe locations are shown in Figures 6A and 6B. PRF temperature measurements during the thermometry stage are plotted for reference.

**
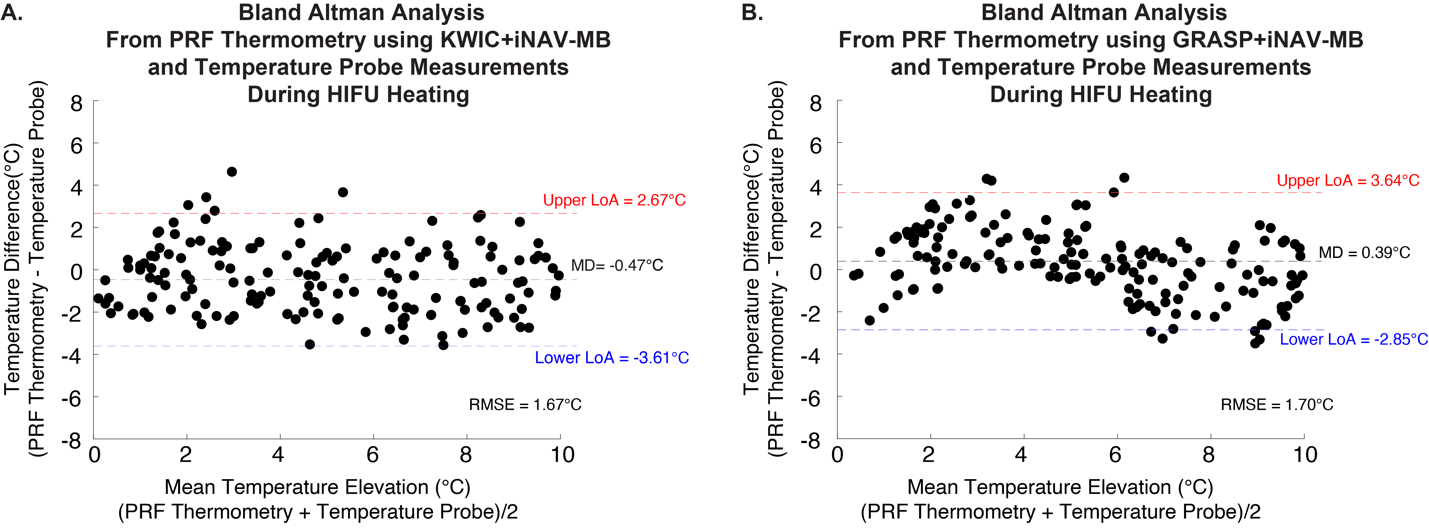
Supporting Information Figure S2.** Bland-Altman plots comparing PRF thermometry measurements using (A) KWIC+ iNAV-MB and (B) GRASP + iNAV-MB at ROI 1 near the HIFU focal point (see Figure 6A) with respect to reference temperature probe readings. The mean difference (MD) and 95% limits of agreement (LoA) are labeled in the plots. In addition, RMS error (RMSE) values are reported.
